# Attitudes and knowledge about post-mortem organ donation among medical students, trainee nurses and students of health sciences in Germany: A cross-sectional study

**DOI:** 10.1007/s00101-020-00812-8

**Published:** 2020-07-21

**Authors:** E. Tackmann, P. Kurz, S. Dettmer

**Affiliations:** Institute of Medical Sociology and Rehabilitation Science, Charité—Universitätsmedizin Berlin, Charitéplatz 1, 10117 Berlin, Germany

**Keywords:** Transplantation, Tissue and organ procurement, Germany, Students, medical, Students, nursing, Transplantation, Gewebe- und Organvermittlung, Deutschland, Medizinstudierende, Auszubildende der Krankenpflege

## Abstract

**Objective:**

In 2018 Germany had the lowest rate of post-mortem organ donation in the Eurotransplant network. Healthcare trainees and students will be important advisors on organ donation for patients in the future. This study aimed to examine 1) attitudes and knowledge about post-mortem organ donation, 2) how past transplantation scandals have affected those attitudes and 3) how satisfied respondents were with the knowledge provided on the courses.

**Methods:**

A cross-sectional study was conducted between 20 March and 8 July 2019 at a university hospital and nursing schools in Berlin and Potsdam, Germany. Study participants were 209 medical students, 106 health sciences students and 67 trainee nurses.

**Results:**

Of the respondents 29.3 and 50.8% knew the tasks of the German Organ Transplantation Foundation and Eurotransplant, respectively. All brain death questions were correctly answered by 56.3% of the medical students, 25.7% of the health sciences students and 50.9% of the trainee nurses (Fisher’s exact test *p* < 0.001, Cramer’s V = 0.242). Transplantation scandals had damaged attitudes towards organ donation for 20.7% of the medical students, 33.3% of the health sciences students and 13.6% of the trainee nurses (χ^2^-test *p* = 0.001, Cramer’s V = 0.164). Asked whether post-mortem organ donation was sufficiently addressed in their courses, 39.5% of the medical students, 60.4% of the health sciences students and 51.9% of the trainee nurses said this was not or tended not to be the case (Kruskal-Wallis H-test *p* < 0.001, Spearman’s rho r = −0.112).

**Conclusion:**

Given the knowledge gaps identified and the respondents’ dissatisfaction with the knowledge they received, organ donation should be better integrated into curricula and training programs.

## Abstract (de)

**Fragestellung:**

Deutschland hatte im Jahr 2018 die niedrigste postmortale Organspenderate im Eurotransplant-Verbund. Auszubildende und Studierende im Gesundheitswesen als zukünftige Ansprechpartner für Patienten haben eine wichtige Beratungsfunktion im Organspendeprozess. Daher ist es Ziel dieser Arbeit, (1) Haltungen und Wissen in Bezug auf postmortale Organspende, (2) den Einfluss vergangener Transplantationsskandale auf Haltungen zu Organspende und (3) die Zufriedenheit mit der Wissensvermittlung unter Auszubildenden und Studierenden zu untersuchen.

**Methoden:**

In diese Querschnittstudie wurden Studierende der Humanmedizin (erstes bis drittes und neuntes bis zwölftes Semester) und Studierende der Gesundheitswissenschaften sowie Auszubildende der Gesundheits- und Krankenpflege einbezogen. Insgesamt haben 209 Medizinstudierende, 106 Studierende der Gesundheitswissenschaften (GeWi) und 67 Auszubildende der Gesundheits- und Krankenpflege (GKP) im Zeitraum vom 20. März bis 08. Juli 2019 an der Studie teilgenommen. Die Daten wurden mittels Paper-Pencil- und Online-Fragebogen erhoben.

**Ergebnisse:**

Von den Befragten konnten 29,3 % sowie 50,8 % die Aufgaben der Deutschen Stiftung Organtransplantation und von Eurotransplant richtig benennen. Alle Hirntod-bezogenen Fragestellungen wurden von 56,3 % der Medizinstudierenden, 25,7 % der GeWi-Studierenden und 50,9 % der GKP-Auszubildenden richtig beantwortet (exakter Test nach Fisher *p* < 0,001, Cramer-V = 0,242). Insgesamt 57,6 % aller Befragten gaben an, ein erweitertes Opt-out-System zu bevorzugen, nur 12,1 % gaben das in Deutschland bestehende erweiterte Opt-in-System an. In einem erweiterten Opt-in-System erfolgt eine Organspende nach Zustimmung der verstorbenen Person, liegt diese nicht vor, sind die nächsten Angehörigen entscheidungspflichtig. In einem Opt-out-System wird die Zustimmung der verstorbenen Person vorausgesetzt, eine Ablehnung wird demnach mündlich oder schriftlich festgehalten. Insgesamt 20,7 % der Medizinstudierenden, 33,3 % der GeWi-Studierenden und 13,6 % der GKP-Auszubildenden geben an, dass ihre Haltung zur Organspende aufgrund der Transplantationsskandale negativ beeinflusst wurde (Chi-Quadrat-Test *p* = 0,001, Cramer-V = 0,164); 39,5 % der Medizinstudierenden, 60,4 % der GeWi-Studierenden und 51,9 % der GKP-Auszubildenden sind nicht oder eher nicht der Meinung, dass postmortale Organspende ausreichend in der Ausbildung/im Studium thematisiert wird (H-Test nach Kruskal-Wallis *p* < 0,001; Spearman-Rho r = −0,112).

**Diskussion:**

In Anbetracht der erhobenen Wissenslücken und der Unzufriedenheit mit der Wissensvermittlung erscheint eine verbesserte Integration des Themas Organspende in die Curricula und Ausbildungspläne notwendig. Im Nationalen Kompetenzbasierten Lernzielkatalog Medizin wird die Vermittlung von ethischen und therapeutischen Aspekten der Organtransplantation sowie von unterschiedlichen Todesdefinitionen im Rahmen des Medizinstudiums bereits empfohlen.

## Treten Sie in den Austausch

Diese Arbeit einer deutschsprachigen Autorengruppe wurde für *Der Anaesthesist* in Englisch eingereicht und angenommen. Die deutsche Zusammenfassung wurde daher etwas ausführlicher gestaltet. Wenn Sie über diese Zusammenfassung hinaus Fragen haben und mehr wissen wollen, nehmen Sie gern in Deutsch über die Korrespondenzadresse am Ende des Beitrags Kontakt mit den Autoren auf. Die Autoren freuen sich auf den Austausch mit Ihnen.

## Introduction

Between 2010 and 2018, the number of post-mortem organ donors in Germany fell from 1217 to 933 [7]. Despite past transplantation scandals at German transplantation centers being widely discussed in the media, acceptance for post-mortem organ donation among the general population remains as high as ever [11]. The number of people holding organ donor cards in Germany has risen from between 11 and 12% in the period 1999–2003 (Forsa [1999; 2], *N* = 1,003, German survey, respondents older than 18; Forsa [2001; 2], *N* = 3,254, German survey, respondents older than 14; Forsa [2003; 2], *N* = 1,001, German survey, respondents between the ages of 14–24 [2]) to 36% in 2018 (figures from the Federal Center for Health Education, Bundeszentrale für gesundheitliche Aufklärung [BZgA], *N* = 4001). The majority of respondents in the BZgA survey were in favor of their organs being donated after death [2, 3, 11]. Nevertheless, with 11.3 post-mortem organ donors per million people in 2018, Germany has the lowest donation rate in the Eurotransplant network, which covers Belgium, the Netherlands, Luxembourg, Germany, Austria, Slovenia, Croatia and Hungary [8]. In Germany, the German Organ Transplantation Foundation (DSO) is responsible for coordinating the organ donation process, while Eurotransplant manages organ allocation and the waiting list [19, 20].

The type of organ donation system affects attitudes to organ donation. In countries with an opt-in system, organ donation can be perceived as more altruistic than in opt-out countries, where consent to organ removal is presumed and non-consent must be recorded in writing [6]. Opt-out countries have higher post-mortem organ donation rates than opt-in countries [14, 18]. In 1997, Germany legally established an extended opt-in system which requires consent from potential donors in the form of an organ donor card or living will. If a person dies without having documented their consent in this way, the family will be asked to decide on their behalf. Under the decision model adopted in 2012, people in Germany receive postal information about organ donation and an organ donor card, from their health insurance provider every 2 years [13, 17, 19].

Germany’s organ shortage is partly the result of problems experienced by hospitals in identifying donors and reporting them to the relevant authority. Although an analysis by Schulte et al. [16] of German donor data collected between 2010 and 2015 found that the number of potential organ donors had risen, it also found that the contact quotient regarding organ donation had declined [16].

In light of this, healthcare students and trainees must be informed at an early stage about the practical aspects and problems of post-mortem organ donation in Germany. In 2013, a nationwide survey of medical students (*N* = 1370) found that 75.8% of respondents carried an organ donor card. These students were more trusting and less fearful of organ donation and the organ donation system than students who did not report owning an organ donor card [22]. A 2002 survey of medical students and doctors in Freiburg found that knowledge about post-mortem organ donation increased as students advanced through their education [15]. A study conducted in the UK in 2000 found no differences in attitudes to organ donation between nursing students and medical students (*N* = 72) [4]. In a survey of medical staff potentially involved in the organ donation process (*N* = 2983) at Bavarian hospitals, nursing staff were less willing to donate (66%) than doctors (82%). In addition, 28% of respondents said that the transplantation scandals had negatively affected their attitudes to post-mortem organ donation [10].

To our knowledge, no comparative study has been carried out in Germany on attitudes and knowledge about post-mortem organ donation among medical students, trainee nurses and health sciences students, who will be the future contacts for organ donation and will help to drive healthcare promotion and research. We therefore designed the present study to identify differences among the abovementioned students and trainees regarding: 1) their attitudes and knowledge about post-mortem organ donation in Germany, 2) how past transplantation scandals have affected their attitudes to organ donation and 3) their satisfaction with the knowledge that their study or training programs provide about post-mortem organ donation.

## Material and methods

A survey of students studying medicine or for a bachelor of arts in health sciences at the Charité—Universitätsmedizin Berlin, and trainee nurses from participating nursing schools was carried out between 20 March and 8 July 2019 via online and paper and pencil questionnaires. People who were training to be nurses or studying medicine or health sciences in Berlin or Potsdam and were at least 16 years of age were included in the study. Regarding the medical students, the study included both new students (in semesters 1–3) and those approaching graduation (in semesters 9–12) in order to compare their levels of knowledge. Those who were under 16 years of age, studying or training outside Berlin or Potsdam, or did not agree to participate were excluded. The following nursing schools participated in the survey: Wannsee-Schule e. V. Schule für Gesundheitsberufe, biz Bildungszentrum für Pflegeberufe der DRK-Schwesternschaft Berlin e. V., Alexianer Akademie für Gesundheitsberufe Berlin/Brandenburg, Gesundheitsakademie der Charité—Universitätsmedizin Berlin, Evangelisches Zentrum für Altersmedizin.

Before the survey began, 39 participants completed an online pretest between 24 January and 6 March 2019. The online survey took place between 20 March and 31 May 2019, and the paper and pencil survey took place between 1 June and 8 July 2019. In total, 67 trainee nurses, 106 students in the health sciences bachelors program, and 209 medical students met the inclusion criteria (*N* = 382).

**Fig. 1:**
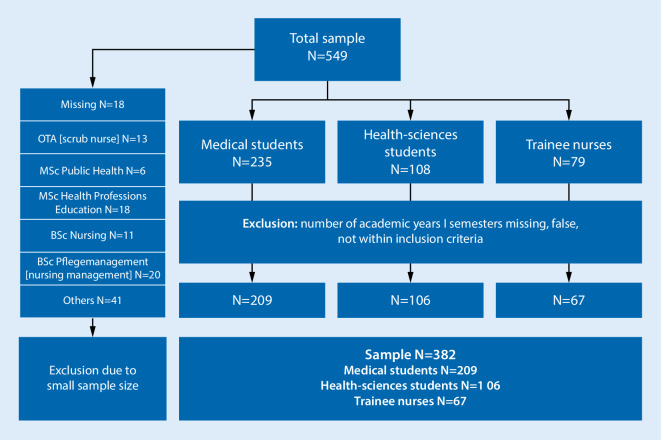
Flow chart of the study population. *MSc* Master of Science, *BSc* Bachelor of Science, *OTA* Operationstechnische/r Angestellte/r, srub nurse

A total of 12 trainee nurses, 26 medical students and 2 health sciences students were excluded from the study because their semester data were missing, false (number of semesters exceeded the standard program duration), or did not meet the inclusion criteria. Other healthcare students and trainees (scrub nurses, students on an MSc in public health or in health professions education) at the Charité—Universitätsmedizin Berlin and other universities and nursing schools in Berlin who were initially included were later excluded because the sample size was too small for statistical analysis (see Fig. 1). The sociodemographic characteristics of the study participants are shown in Table 1, grouped according to degree/training program.

**Table 1:** Sociodemographic characteristics of the study participants (*N* = 382)

	Medical students (*N* = 209)	Health sciences students (BSc) (*N* = 106)	Trainee nurses (*N* = 67)
Age (mean ± SD)	24.5 ± 4.4 years Missing *N* = 2	30.3 ± 4.4 years Missing *N* = 2	22.2 ± 3.9 years Missing *N* = 1
Gender	Female 74.6%	Female 82.1%	Female 85.1%
	Male 25.4%	Male 16.0%	Male 14.9%
		Other 1.9%	
Nationality	German 89.5%	German 97.2%	German 89.6%
	Other 10.5%	Other 2.8%	Other 10.4%
Marital status	Married 9.6%	Married 12.3%	Married 4.5%
	In a partnership 38.8%	In a partnership 63.2%	In a partnership 38.8%
	Single 51.7%	Single 24.5%	Single 56.7%
Religion	Roman Catholic 16.9%	Roman Catholic 15.1%	Roman Catholic 10.4%
	Protestant 30.0%	Protestant 19.8%	Protestant 22.4%
	Sunni 1.0%	None 60.4%	None 64.2%
	None 51.2%	Other 4.7%	Other 3.0%
	Other 1.0%		
	Missing *N* = 2		
School leaving qualification	MSA/Realschulabschluss^a^ 0.5%	MSA/Realschulabschluss^a^ 1.9%	Hauptschulabschluss^a^ 1.5%
	Abitur^a^ 94.7%	Fachabitur^a^ 3.8%	MSA/Realschulabschluss^a^ 35.8%
	Other 4.8%	Abitur^a^ 92.4%	Fachabitur^a^ 7.5%
	Missing *N* = 1	Other 1.9%	Abitur^a^ 55.2%
		Missing *N* = 1	
Training year/semester	Start of course 1st semester 1.9% 2nd semester 27.8% 3rd semester 17.2%	1st semester 0.9%	1st year 20.9%
		2nd semester 28.3%	2nd year 58.2%
		3rd semester 3.8%	3rd year 20.9%
		4th semester 17.0%	
	End of course 9th semester 6.2% 10th semester 19.6% Practical year 27.3%	5th semester 1.9%	
		6th semester 48.1%	

^*a*^*MSA/Realschulabschluss* intermediate school leaving certificate, *Abitur* university entrance qualification, *Fachabitur* subject-specific university entrance qualification, *Hauptschulabschluss* general school leaving certificate

Data analysis was performed with SPSS Statistics 25 (IBM, Armonk, NY, USA). A number of group comparisons, correlation analyses, and tests for normal distribution were carried out in this exploratory study. In what follows, *p*-values were specified as exploratory and as significant at ≤0.05; they should not, however, be interpreted as confirmatory. To ascertain group differences, the χ^2^-test was used for nominal characteristics with sufficient cell size, and Fisher’s exact test was used for those with small cell sizes. For ordinal variables, normal distribution was excluded using the Shapiro-Wilk test, and then the Kruskal-Wallis H-test was applied. The degree of association (correlation) was measured using Cramer’s V for nominal characteristics, and Spearman’s rho for ordinal variables. A correlation measured with a Cramer’s V of <0.20 was described as a very low association, 0.2–0.5 as a low association, and 0.5–0.7 as a medium association [23]. A Spearman’s rho of r = 0.1 corresponded to a small effect size, r = 0.3 to a medium effect size, and r = 0.5 to a large effect size (see Table 2; [5]).

**Table 2:** Correlations (Spearman’s rho and Cramer’s V), with Kruskal-Wallis H test *p* ≤ 0.05, χ^2^-test ≤0.05 or Fisher’s exact test ≤0.05

Comparison of degree and training programs									
		Attitudes	Recorded in writing	Electronic health card	Subjective knowledge	Eurotransplant	DSO	Knowledge of regulation	Preferred regulation
Training/degree	Correlation coefficient	0.037	0.220**	0.163**	0.067	0.245**	0.190*	0.273**	0.142*
–	*N*	355	351	345	344	329	334	336	335
–	–	Trust in healthcare system	Trust in organ donation system	Trust regarding brain death	Attitude after transplantation scandals	Education about organ donation	Knowledge of organ donation process	Knowledge of organ donation law	Brain-death score
Training/degree	Correlation coefficient	−0.002	0.016	−0.016	0.164**	−0.112*	−0.011	−0.034	0.242**
–	*N*	339	329	337	345	330	325	326	332
*Brain death score*					*Health sciences degree*				
–	–	Reaction	Coma	Persistent vegetative state	–	–	Subjective knowledge	Knowledge of organ donation process	Knowledge of organ donation law
Training/degree	Correlation coefficient	0.193**	0.118	0.138*	Start and end of studies	Correlation coefficient	0.217*	0.285**	0.210*
–	*N*	335	335	333	–	*N*	102	99	100
*Medical degree*							*Nursing training*		
–	–	Attitudes	Subjective knowledge	Knowledge of organ donation process	Knowledge of the concept of brain death	Knowledge of organ donation law	–	–	Education about organ donation
Start and end of studies	Correlation coefficient	0.185*	0.438**	0.435**	0.570**	0.320**	Training year	Correlation coefficient	−0.349*
–	*N*	192	185	176	176	176	–	*N*	52

* *p* ≤ 0.05, ** *p* ≤ 0.01

The participants’ attitudes and knowledge were mostly assessed using a 5-point Likert scale (ranging from either negative to positive, or disagree to agree). More information on the questionnaire are given in Table 3. Missing answers were not included. For all knowledge questions, the answer “don’t know” was included in the statistical analysis and presented in this paper. For the other questions, “don’t know” was only included if it accounted for >5% of all answers. This study was approved by the university ethics committee. Participation was voluntary.

**Table 3:** Excerpt of the questionnaire

No.	Questions	Possible answers
18	What is your view of post-mortem organ donation? *Wie stehen Sie grundsätzlich zur postmortalen Organspende?*	5‑point Likert scale from negative to positive and don’t know
23	I would agree to post-mortem organ donation on behalf of my relatives. *Ich würde einer postmortalen Organspende bei meinen Angehörigen zustimmen*	5‑point Likert scale from disagree to agree and don’t know
24	Would you donate your organs after your death? *Würden Sie selbst nach Ihrem Tod Organe spenden?*	Yes, no and don’t know
27	Have you recorded your attitude towards post-mortem organ donation in writing? *Haben Sie Ihre Haltung zur Organspende schriftlich festgehalten?*	Yes and no
32	Would you record your attitude on your electronic health card? *Würden Sie ihre Haltung zur Organspende auf der Krankenversicherungskarte festhalten wollen?*	Yes, no, don’t know and “I don’t own an electronic health card”
33	How would you rate your knowledge about post-mortem organ donation? *Wie schätzen Sie Ihr Wissen über postmortale Organspende ein?*	5‑point Likert scale from bad to good and don’t know
35	I have trust in the German healthcare system. *Ich habe Vertrauen in das deutsche Gesundheitssystem*	5‑point Likert scale from disagree to agree and don’t know
36	I have trust in the German organ donation system. *Ich habe Vertrauen in das deutsche Organspendesystem*	5‑point Likert scale from disagree to agree and don’t know
37	I have trust in doctors determining brain death reliably. *Ich habe Vertrauen in die sichere Feststellung des Hirntodes*	5‑point Likert scale from disagree to agree and don’t know
40	Did the transplantation scandals negatively affect your attitude towards organ donation? *Haben die vergangenen Transplantationsskandale in Deutschland einen negativen Einfluss auf ihre Haltung zur Organspende gehabt?*	Yes, no and don’t know
42	Which institution is responsible for organ allocation and waiting lists in Germany? *Welche Institution ist in Deutschland für die Organverteilung und Wartelistenführung verantwortlich?*	DSO Bundesärztekammer Krankenhäuser Transplantationszentren Bundesministerium für Gesundheit Eurotransplant Robert-Koch-Institut don’t know
43	Which institution is responsible for organ donation coordination in Germany? *Welche Institution ist in Deutschland für die Koordination der Organspende verantwortlich?*	DSO Bundesärztekammer Krankenhäuser Transplantationszentren Bundesministerium für Gesundheit Eurotransplant Robert-Koch-Institut don’t know
44	Please select below, which type of organ donation system is in place in Germany. *Bitte geben Sie an, welche gesetzliche Regelung momentan in Deutschland gültig ist*	Opt-out system Extended opt-out system Opt-in system Extended opt-in system *In the questionnaire, answers are given in descriptive form*
45	Which type of organ donation system would you prefer? *Welche gesetzliche Regelung bevorzugen Sie?*	Opt-out system Extended opt-out system Opt-in system Extended opt-in system *In the questionnaire, answers are given in descriptive form*
**Brain death score**		
52	Someone who is brain dead can breathe without the support of a ventilator. *Eine hirntote Person kann ohne die Unterstützung eines Beatmungsgeräts atmen*	Yes, no and don’t know
53	Someone who is brain dead can ever wake up (recover). *Eine hirntote Person kann jemals wieder aufwachen (sich erholen)*	Yes, no and don’t know
54	Someone who is brain dead can react (grimace, move away, or blink) if someone touches their eyeball. *Eine hirntote Person kann reagieren (grimassieren, sich wegbewegen, mit dem Auge zwinkern), wenn jemand ihren Augapfel berührt*	Yes, no and don’t know
55	A person can be brain dead even if their heart is still beating. *Eine Person kann hirntot sein, wenn ihr Herz noch schlägt*	Yes, no and don’t know
56	Brain death is different from a coma. *Der Hirntod unterscheidet sich von einem Koma*	Yes, no and don’t know
57	Brain death is different from a vegetative state. *Der Hirntod unterscheidet sich von einem permanenten vegetativen Zustand (“Wachkoma”)*	Yes, no and don’t know
**Knowledge**		
66	In my opinion, organ donation is addressed sufficiently in my courses. *Organspende wird meiner Meinung nach ausreichend in meinem Studium/meiner Ausbildung thematisiert*	5‑point Likert scale from disagree to agree and don’t know
67	In my opinion, brain death is addressed sufficiently in my courses. *Hirntod wird meiner Meinung nach ausreichend in meinem Studium/meiner Ausbildung thematisiert*	5‑point Likert scale from disagree to agree and don’t know
68	I can explain the process of post-mortem organ donation to a patient or someone who is interested. *Ich fühle mich dazu befähigt, einer Patientin/einem Patienten oder Interessierten den Verlauf einer Organspende zu erklären*	5‑point Likert scale from disagree to agree and don’t know
69	I can explain the brain death concept to a patient or someone who is interested. *Ich fühle mich dazu befähigt, einer Patientin/einem Patienten oder Interessierten das Konzept des Hirntods zu erklären*	5‑point Likert scale from disagree to agree and don’t know
70	I can explain the legal foundation of organ donation in Germany to a patient or someone who is interested. *Ich fühle mich dazu befähigt, einer Patientin/einem Patienten oder Interessierten die gesetzlichen Grundlagen der Organspende in Deutschland zu erklären*	5‑point Likert scale from disagree to agree and don’t know

## Results

### Comparison of attitudes and knowledge among the students and trainees

**Fig. 2:**
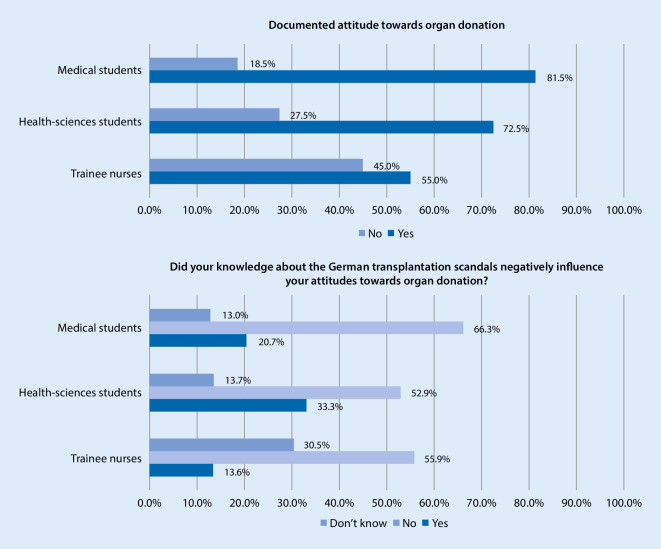
Documented attitudes towards organ donation (*N* = 189, *N* = 102, *N* = 60) and influence of transplantation scandals on attitudes (*N* = 184, *N* = 102, *N* = 59)

The majority of survey participants had a positive view of post-mortem organ donation (72.7% answered positive, 18.0% answered mostly positive; *N* = 192, *N* = 102, *N* = 61). Medical students in semesters 9–12 had a significantly more positive attitude to post-mortem organ donation than those just starting their studies (the answer positive rose from 73.9% to 89.0%; Kruskal-Wallis H test *p* = 0.011, Spearman’s rho r = 0.185; *N* = 192). Attitudes to organ donation among the health sciences students and trainee nurses did not change significantly during their studies or training (*N* = 102, *N* = 61). A total of 86.5% of survey participants said they would donate organs after their death, with no significant difference between the groups (4.5% answered no , 9.0% answered don’t know; *N* = 191, *N* = 103, *N* = 61); however, just 81.5% of the medical students, 72.5% of the health sciences students and 55.0% of the trainee nurses had recorded their attitude in writing (χ^2^-test *p* < 0.001, Cramer’s V = 0.220; *N* = 189, *N* = 102, *N* = 60) (see Fig. 2). In total, 59.2% of the medical students, 74.5% of the health sciences students and 64.4% of the trainee nurses said they would record their attitude on their electronic health card (14.5% answered don’t know, 4.3% answered I don’t have an electronic health card; Fisher’s exact test *p* = 0.004, Cramer’s V = 0.163; *N* = 184, *N* = 102, *N* = 59). In terms of agreeing to post-mortem organ donation on behalf of relatives, 63.3% were in favor, and 19.8% were mostly in favor, with no significant difference between the groups (*N* = 186, *N* = 99, *N* = 58).

Among the medical students, 32.4% and 23.8% rated their knowledge about post-mortem organ donation as mostly good or good, respectively. This was compared to 27.5% and 17.6% of the health sciences students, and 21.1 and 7.0% of the trainee nurses (*N* = 185, *N* = 102, *N* = 57). Subjective assessments of knowledge differed significantly between medical students at the start and end of their studies (the answer good rose from 3.4% to 42.3%; Kruskal-Wallis H test *p* < 0.001, Spearman’s rho r = 0.438; *N* = 185). The same was true of the health sciences students (the answer good rose from 9.1% to 21.7%; Kruskal-Wallis H test *p* = 0.029, Spearman’s rho r = 0.217; *N* = 102). Subjective assessments of knowledge among the trainee nurses did not change significantly during their training (*N* = 59).

A total of 58.7% of the medical students (*N* = 179), 54.3% of the health sciences students (*N* = 94) and 19.6% of the trainee nurses (*N* = 56) named Eurotransplant as the institution responsible for organ allocation and waiting lists (Fisher’s exact test *p* < 0.001, Cramer’s V = 0.245). The answer don’t know was given by 28.6% of all respondents and 42.9% of the trainee nurses. Similarly, 34.6% of the medical students (*N* = 179), 21.2% of the health sciences students (*N* = 99) and 26.8% of the trainee nurses (*N* = 56) said that the German Organ Transplantation Foundation was responsible for coordinating organ transplantations (Fisher’s exact test *p* = 0.013, Cramer’s V = 0.190). In total, 33.8% of the survey participants and 48.2% of the trainee nurses answered don’t know.

Regarding the extended opt-in system that exists in Germany, 88.8% of the medical students (*N* = 178), 86.3% of the health sciences students (*N* = 102) and 53.6% of the trainee nurses (*N* = 56) were able to name it correctly (1.8% answered don’t know; Fisher’s exact test *p* < 0.001, Cramer’s V = 0.273). Of all the respondents, 57.6% said they would prefer an extended opt-out system, while just 12.1% preferred the current extended opt-in system (Fisher’s exact test *p* = 0.033, Cramer’s V = 0.142; medical students: 64.6%, *N* = 178; health sciences students: 45.5%, *N* = 100; trainee nurses: 51.8%, *N* = 52).

**Fig. 3:**
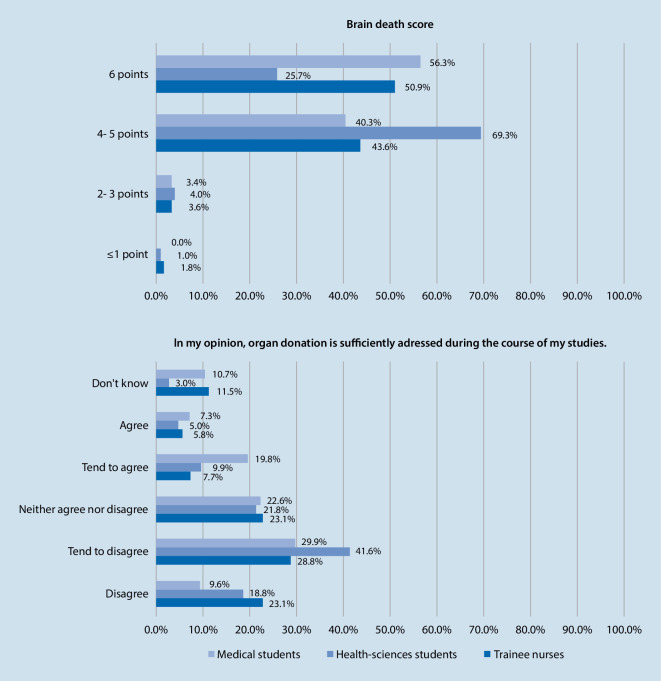
Brain death score (*N* = 176, *N* = 101, *N* = 55) and representation of organ donation in degree or training programmes (*N* = 177, *N* = 101, *N* = 52)

To evaluate knowledge about brain death, a score by Tawil et al. [21] was adapted and included six general, closed questions [21]. A total of 12.5% of the respondents did not say that a brain dead person cannot breathe without a ventilator (8.7% answered yes , 3.9% answered don’t know ; *N* = 178, *N* = 102, *N* = 55). A further 5.7% were of the opinion that a brain dead person can wake up and recover, while 5.1% answered don’t know (*N* = 178, *N* = 102, *N* = 55). Asked whether a brain dead person can react (grimace, pull away), 22.1% said they could (15.2% of the medical students, 34.3% of the health-sciences students, 21.8% of the trainee nurses), and 11.9% did not know the answer (*N* = 178, *N* = 102, *N* = 55; χ^2^-test *p* < 0.001, Cramer’s V = 0.193). A total of 3.9% of respondents said that a person cannot be brain dead if the heart is still beating, while 4.2% did not know the right answer (*N* = 178, *N* = 101, *N* = 55). A further 5.4% and 9.3% did not know that brain death differs to a coma or persistent vegetative state (1.5% and 3.3% answered no, 3.9% and 6.0% answered don’t know; *N* = 178, *N* = 102, *N* = 55). In total, 56.3% of the medical students, 25.7% of the health-sciences students and 50.9% of the trainee nurses who answered all the questions gave correct answers to all the questions related to brain death (Fisher’s exact test *p* < 0.001, Cramer’s V = 0.242; *N* = 176, *N* = 101, *N* = 55) (see Fig. 3).

### Trust in the healthcare and organ donation systems

A total of 28.3% and 41.7% of the medical students trusted or mostly trusted the German healthcare system. The same was true for 9.8% and 32.4% of the health sciences students, and for 5.3% and 19.3% of the trainee nurses (*N* = 180, *N* = 102, *N* = 57). Taken together, 23.1% and 36.8% of all respondents said that they trusted or mostly trusted the organ donation system (*N* = 176, *N* = 97, *N* = 56).

When asked about doctors determining brain death, 78.7% of the survey participants said they trusted or mostly trusted them to do this reliably (*N* = 181, *N* = 99, *N* = 57). Asked whether the transplantation scandals had negatively affected their attitudes, 23.2% said yes (20.7% of the medical students, 33.3% of the health sciences students, 13.6% of the trainee nurses). A further 16.2% (13.0% of the medical students, 13.7% of the health sciences students, 30.5% of the trainee nurses) answered don’t know to this question (χ^2^-test *p* = 0.001, Cramer’s V = 0.164; *N* = 184, *N* = 102, *N* = 59) (see Fig. 2).

### Satisfaction with knowledge provided during studies or training

A total of 39.5% of the medical students, 60.4% of the health sciences students and 51.9% of the trainee nurses were not or mostly not of the opinion that post-mortem organ donation is sufficiently addressed by their degree or training programmes (8.5% answered don’t know; Kruskal-Wallis H test *p* < 0.001, Spearman’s rho r = −0.112; *N* = 177, *N* = 101, *N* = 52) (see Fig. 3). Just 28.3% of respondents agreed or tended to agree that they could explain the process of post-mortem organ donation, and 46.5% disagreed or tended to disagree (*N* = 176, *N* = 99, *N* = 50). In addition, 46.6% of respondents disagreed or tended to disagree that they could explain the legal foundations of post-mortem organ donation (*N* = 176, *N* = 100, *N* = 50).

Medical students at the beginning and end of their studies did not differ significantly in their satisfaction with the knowledge that their programs provide about organ donation (*N* = 177); however, the students at the end of their studies rated themselves as significantly more able to explain the process of post-mortem organ donation (Kruskal-Wallis H test *p* < 0.001, Spearman’s rho r = 0.435, *N* = 176), the concept of brain death (Kruskal-Wallis H test *p* < 0.001, Spearman’s rho r = 0.570, *N* = 176) and the legal foundations of organ donation (Kruskal-Wallis H test *p* < 0.001, Spearman’s rho r = 0.320, *N* = 176).

Similarly, health sciences students at the start of their bachelor’s (semesters 1–3) and at the end (semesters 4–6) did not differ in their levels of satisfaction regarding the knowledge provided about organ donation and in their assessments of their knowledge about the concept of brain death. They did, however, differ significantly in terms of how they assessed their ability to explain the legal foundations and the process of organ donation (Kruskal-Wallis H test *p* = 0.037, Spearman’s rho r = 0.210, *N* = 100; Kruskal-Wallis H test *p* = 0.005, Spearman’s rho r = 0.285, *N* = 99).

Regarding the trainee nurses, satisfaction with the knowledge provided about organ donation differed significantly depending on the number of years in training, and a negative correlation was identified (Kruskal-Wallis H test *p* = 0.021, Spearman’s rho r = −0.349, *N* = 52). Of the nurses in their first training year (*N* = 12), 41.7% answered don’t know, and 41.7% gave an average satisfaction rating. Of the trainees in their second (*N* = 29) and third (*N* = 11) year, 62.0% and 63.7%, respectively, disagreed or tended to disagree that their training program sufficiently addressed post-mortem organ donation. No significant differences existed between the training years regarding self-assessments of the ability to explain the process of post-mortem organ donation, the concept of brain death, and the legal foundations.

## Discussion

Active and passive acceptance for post-mortem organ donation is high among healthcare students and trainees in Germany. The majority have also recorded their attitude in writing and would document their attitude on their electronic health card. The majority would also agree to organ donation on behalf of family members. Only roughly half of the respondents were able to correctly name the German Organ Transplantation Foundation (DSO) as the institution responsible for coordinating organ donation. Nearly half of the respondents were dissatisfied with the amount of knowledge provided about post-mortem organ donation, and no positive change occurred at later stages in their degree/training programs. Over half of the medical students and trainee nurses were in favor of an opt-out system being introduced. Overall, medical students outperformed the other two groups on the knowledge questions. A minority in all three groups said that the transplantation scandals had changed their attitudes to post-mortem organ donation, while 16.2% responded with don’t know.

Terbonssen et al. [22] identified a high willingness among medical students to document their attitudes to post-mortem organ donation in the form of an organ donor card [22]. The representative survey that the BZgA carried out for the general population in Germany in 2018 found that 39% had documented their attitude in writing. A further 84% reported a positive view of post-mortem organ donation, and 54% said they would agree to organ donation on behalf of a deceased relative [3]. The groups included in the present study all showed a comparably frequent positive or generally positive opinion, had more frequently recorded their attitude in writing, and would mostly agree to an organ donation on behalf of a deceased relative. The BZgA used closed questions for its survey, while the present survey used a 5-point Likert scale.

The study by Tawil et al. [21] found that just 33% of the medical students surveyed could answer all 5 brain death questions correctly. The last question on differentiating between a coma and a persistent vegetative state was divided into two questions for the present survey. The medical students and the trainee nurses gave the right answers more frequently than the respondents in the previous sample [21]; however, only roughly half of all respondents answered all the questions correctly. This highlights the need for a greater focus on the topic of brain death.

## Conclusion

To our knowledge, the present survey is the first time that attitudes and knowledge among healthcare students and trainees have been compared in a German-speaking country. Deficits in knowledge about the way organ donations are organized and about aspects of brain death were identified among the health sciences students and trainee nurses in particular. Dissatisfaction with the provision of content about organ donation was primarily expressed by these two groups.

Course content on this topic should be expanded and/or deepened in order to prevent misconceptions and make it easier to communicate with patients and relatives on this topic. Particularly among the trainee nurses, the survey showed no subjective increase in knowledge as training progressed. Regarding medical students, the German national competence-based catalogue of learning objectives in medicine states that medical students should know different definitions of death and apply those in a clinical setting. Furthermore, they should be familiar with clinical and ethical implications of organ transplantation [12]. More than one third of included medical students were dissatisfied or relatively dissatisfied with the provision of content about organ donation at medical school.

To improve satisfaction, standardized patients can be used to teach social, ethical and medical aspects of organ donation and brain death [1]. Interventions such as lectures on the organ donation process, donor eligibility and policies including a small group discussion with standardized patients have been proven to significantly increase knowledge on organ donation [9].

All groups surveyed were in favor of introducing an opt-out system and would record their attitude to post-mortem organ donation on their electronic health card. The survey participants generally had a positive view of these potential changes in the law. In light of these documented views and a corresponding potential increase in organ donation rates, these aspects should continue to be discussed by German lawmakers and in the media.

## Limitations

The small sample size means that the data collected are not representative of all students and trainees in Berlin. The universities and nursing schools did not provide sociodemographic data about their trainees and students, which makes it impossible to compare the sample with the population. It was also not possible to randomize the inclusion of the participants because they had to be contacted for the online and paper and pencil surveys.
